# Selection on individuals of introduced species starts before the actual introduction

**DOI:** 10.1111/eva.13159

**Published:** 2020-12-15

**Authors:** Adrián Baños‐Villalba, Martina Carrete, Jose Luis Tella, Julio Blas, Jaime Potti, Carlos Camacho, Moussa Sega Diop, Tracy A. Marchant, Sonia Cabezas, Pim Edelaar

**Affiliations:** ^1^ University Pablo de Olavide Sevilla Spain; ^2^ Estación Biológica de Doñana‐CSIC Sevilla Spain; ^3^ AfriWet Dakar Senegal; ^4^ University of Saskatchewan Saskatoon Canada

**Keywords:** biological invasion, brain size, feather corticosterone, invasiveness, pre‐introduction selection, selective filters, wildlife trade

## Abstract

Biological invasion is a global problem with large negative impacts on ecosystems and human societies. When a species is introduced, individuals will first have to pass through the invasion stages of uptake and transport, before actual introduction in a non‐native range. Selection is predicted to act during these earliest stages of biological invasion, potentially influencing the invasiveness and/or impact of introduced populations. Despite this potential impact of pre‐introduction selection, empirical tests are virtually lacking. To test the hypothesis of pre‐introduction selection, we followed the fate of individuals during capture, initial acclimation, and captivity in two bird species with several invasive populations originating from the international trade in wild‐caught pets (the weavers *Ploceus melanocephalus* and *Euplectes afer*). We confirm that pre‐introduction selection acts on a wide range of physiological, morphological, behavioral, and demographic traits (incl. sex, age, size of body/brain/bill, bill shape, body mass, corticosterone levels, and escape behavior); these are all traits which likely affect invasion success. Our study thus comprehensively demonstrates the existence of hitherto ignored selection acting before the actual introduction into non‐native ranges. This could ultimately change the composition and functioning of introduced populations, and therefore warrants greater attention. More knowledge on pre‐introduction selection also might provide novel targets for the management of invasive species, if pre‐introduction filters can be adjusted to change the quality and/or quantity of individuals passing through such that invasion probability and/or impacts are reduced.

## 1 INTRODUCTION

The worldwide introduction of exotic species is considered a central component of global change that can have severe ecological and socio‐economic impacts (Vitousek et al., 1996, 1997). Since the eradication of invasive populations is costly and often impractical or impossible, much research has been devoted to avoid the success of future invasions (Kolar & Lodge, 2001). This includes the identification of factors that might contribute to successful invasions, like characteristics of the event itself (e.g., introduction effort or propagule number), the biotic and abiotic characteristics of the invaded ecosystem, and the characteristics of the invading species (Catford et al., 2009). Evolutionary processes that might affect invasion are nonetheless rarely considered.

The invasion process typically involves a number of sequential steps (uptake/capture, transport, introduction, establishment and expansion) separated by barriers potentially acting as selective filters that prevent or allow passing from one stage to another (Blackburn et al., 2011). However, most studies focus on the latest establishment and expansion stages, thereby largely neglecting the earliest stages of invasion (capture, transport, and introduction) despite their potential importance (Puth & Post, 2005). In fact, taxonomic biases in capture and transport may determine which species have opportunities to settle and become invasive (Blackburn et al., 2009). Hence, there is a need for a stronger research focus on the initial invasion stages (Briski et al., 2018), as early selective events set the conditions for later stages: variation removed early on is no longer present later on.

Another feature of current research is a dominant focus on the average characteristics of the studied species, thereby ignoring individual variation within potentially invasive species. Such a bias occurs despite the fact that newly established populations have been documented to undergo rapid evolutionary changes in morphological, behavioral, physiological, and life‐history traits (e.g., Blackburn et al., 2009), supporting the hypothesis that individuals do vary significantly in their invasive potential, and that selection acts on this variation. In summary, research to date has generally neglected the study of the earliest stages of the invasion pathway and especially so with regard to individual variation within‐species. For these reasons, several calls have been made to investigate this knowledge gap involving pre‐introduction selection on individual variation (Briski et al., 2018; Carrete et al., 2012; Chapple et al., 2012). Basically, while it has been acknowledged that natural selection acts on invaders after introduction, we have so far ignored that selective processes analogous to natural selection might be acting before actual introduction.

**Figure 1:**
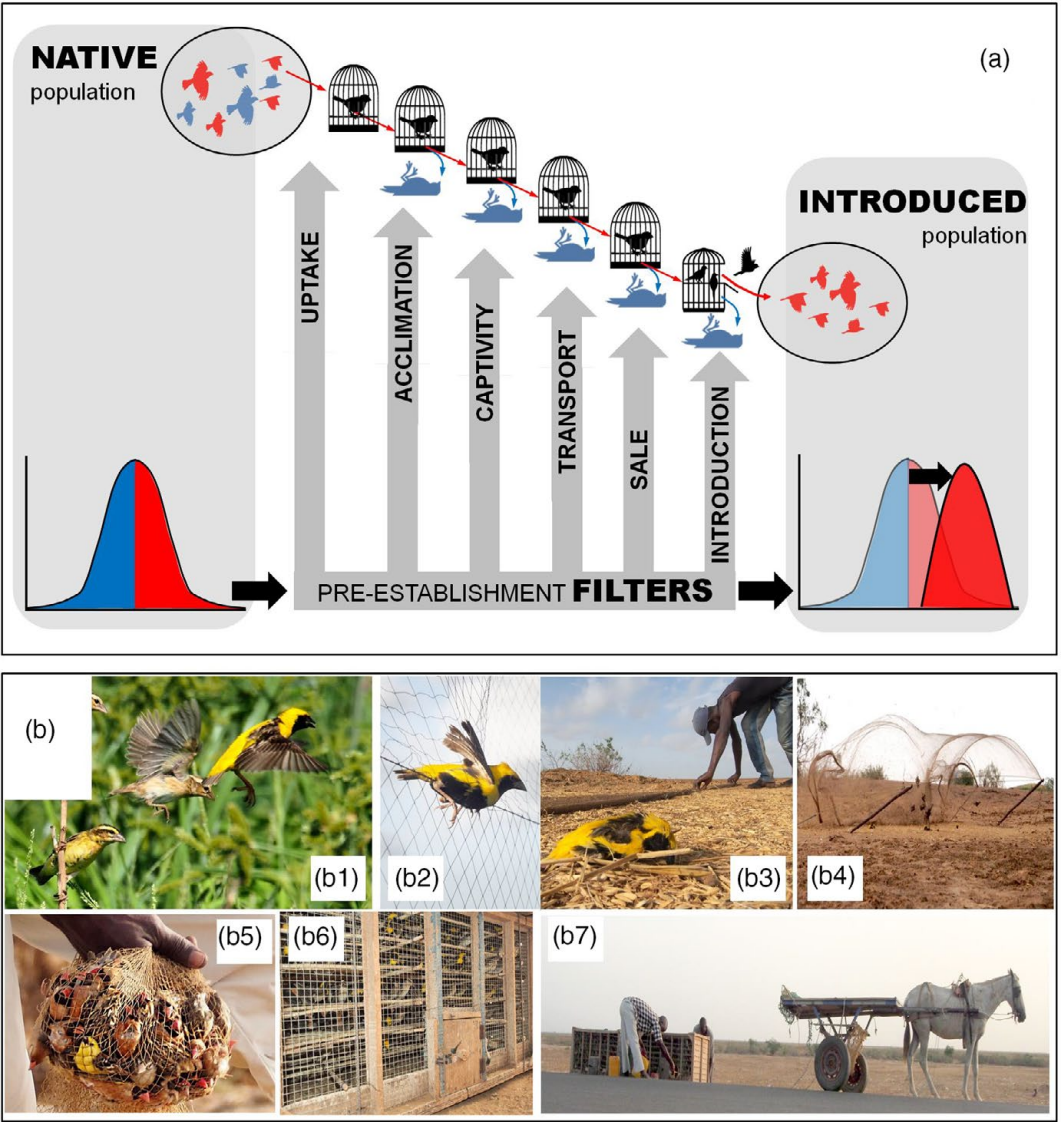
(a) Scheme of potential selective filters acting during the pre‐introduction stages of an invasion process. For each filter, one or more selective pressures eliminate certain individuals from the pool of potential invaders. As a consequence, the invading population (right) is composed of a nonrandom sample from the native population (left) and shows different mean values and frequency distributions of individual traits (here relative frequencies represented with either blue or red colors). (b) Photographic summary of field procedures in Senegal. (b1) A free‐flying flock of native *Euplectes afer* (yellow‐colored birds are males; more dull‐colored birds are females or juveniles). (b2) Presumably less‐ or nonselective capture through mist netting (NATIVE birds in main text). (b3‐b5) Traditional uptake of birds by local Senegalese trappers, using clap nets baited with seeds and stuffed decoys (UPTAKE birds in main text). (b6) Short‐term storage of trapped birds at high densities in traditional cages (Initial acclimation). (b7) Transport of birds. The traditional cages are moved on a horse cart to the nearby road, and then transported 350 km (about 7 hr) to Dakar on the rooftops of public buses (not shown). International export usually takes place from Dakar, after 1–3 months of storage. Photo credits: Julio Blas

Such studies are relevant for several reasons. First, early selection on individual variation could change the composition of the introduced populations (Figure 1a), enhancing or decreasing invasive potential depending on how it acts on relevant traits. Hence, a better knowledge of the early selection process could help us to understand why certain species or introduction events are successful while others are not (Briski et al., 2018; Colautti & Lau, 2015; Zeng et al., 2015). Second, knowing *how* early selection operates might help inform management actions aiming to avoid further invasions (Briski et al., 2018): identified selective filters could be adjusted to reduce the invasiveness of the introduced populations, or even strengthened to block potential invaders completely. Third, many comparative studies implicitly assume that early selection can be ignored. When using average species traits, the values are typically collected from either native or established populations (Blackburn et al., 2009), but these may not reflect accurately the mean of the population during introduction. Other studies try to infer evolution and adaptation in novel ranges via comparison of invasive and native populations, thereby ignoring the possibility that any encountered differences might have arisen during the early, pre‐introduction stages of the invasion, potentially involving no further changes after introduction to the novel range. Hence, as stated before (Briski et al., 2018; Carrete et al., 2012; Chapple et al., 2012), there are various reasons why pre‐introduction selection on individual variation should be no longer ignored.

Here, we tested the hypothesis of pre‐introduction selection in two avian invaders, the Black‐headed Weaver (*Ploceus melanocephalus* Linnaeus 1758) and the Yellow‐crowned Bishop (*Euplectes afer* Gmelin 1789). These African passerines are commonly wild‐caught and subsequently traded as pet birds, leading to the establishment of invasive populations of both species in Europe (Portugal, Spain), and for *Euplectes* also in the Americas (Jamaica, Puerto Rico, Guadalupe) and Asia (Kuwait, United Arab Emirates, Oman) (Abellán et al., 2017; eBird platform, 2020; Lever, 2005; Sanz‐Aguilar et al., 2014). Contrary to the deliberate introductions of the past centuries, many current invasions stem from the international pet‐trade of exotic species. Millions of plants and animals, belonging to hundreds of species, are extracted annually from nature and transported internationally for their trade in pet markets, aquaculture, and gardening (Reaser, 2008). A small portion of these specimens is accidentally or deliberately released or manage to escape, resulting in new invasions. We therefore aimed at testing whether pre‐introduction selection occurs, when it acts, and on which phenotypic traits, by following the fate of a set of individuals previously characterized for various traits across a number of potentially selective events: capture, initial acclimation, and captivity. Previously, we found support for pre‐introduction selection on genetic variation in a dopamine receptor gene (Mueller et al., 2017) related to behavioral variation in *Euplectes afer* (Mueller et al., 2014). As far as we know, this is the only published study investigating pre‐introduction selection on individual variation. In the present study, we expand this earlier work to involve two invasive avian species and a wide array of phenotypic traits, thereby generalizing the focus of Mueller et al. (2017).

We tested for pre‐introduction selection on a number of phenotypic traits involving behavior, physiology, and morphology, traits we a priori thought to be potentially important for invasion success and to be exposed to pre‐introduction selection. Consistent individual variation in behavioral responses to stimuli and situations (Réale et al., 2007) could be important for invasion success, especially when associated with dispersion, social organization, demographic parameters, and energetic demands (Dingemanse et al., 2010; Réale et al., 2007), thereby influencing range expansion into non‐native areas (Liebl & Martin, 2012) or the exploration of novel food resources (Sol et al., 2011). Behavioral variation may be particularly important when facing new and challenging situations, potentially determining the differential survival of individuals (Réale et al., 2007). Corticosterone hormone levels typically elevate in response to increased energetic demands generated under both predictable (e.g., dial rhythms, life cycle) and unpredictable (i.e., stress‐related) situations, and are thus considered major allostasis mediators in birds (Blas, 2015; McEwen & Wingfield, 2003). Variation in both behavior and corticosterone levels might affect survival (Blas et al., 2007; Koren et al., 2012; Rebolo‐Ifran et al., 2015) and potentially explain invasion success (Martin et al., 2017) through selection during pre‐introduction filters. Next, morphology may also be relevant for invasion success and be under selection during the pre‐introduction stages. For example, body size and body mass could be related to capture probability, intra‐ and interspecific competition (Grant, 1968; Leyequién et al., 2006), resource requirements or resistance to temperature changes (Collins et al., 2017; McKechnie & Lovegrove, 2002). Bill traits (incl. size and shape) may be related to feeding ability (e.g., use of new sources of food), thermoregulation, and intra‐ and interspecific competition (Friedman et al., 2019; Grant, 1968; Hasegawa et al., 2015; Hsu et al., 2014). Brain size in birds has been related to the ability to deal with novel situations, capture probability (Møller, 2010), escape strategies (Samia et al., 2015) and the colonization of variable habitats (Fristoe et al., 2017). Finally, the sex, age, and reproductive status of individuals are key components of the demographic composition of a newly introduced population, influencing population survival and growth rate (Barrientos, 2015), and could be under pre‐introduction selection.

## 2 METHODS

### 2.1 Sampling of native populations

Native individuals of *P. melanocephalus* and *E. afer* (NATIVE sample, N*_P. melanocephalus_* = 394, N*_E._* *_afer_* = 446; Figure 1b1) were caught by us using mist nets (Figure 1b2) in September 2014 near the vicinity of Richard Toll, northern Senegal (16°27'45"N−15°42'03"W). According to the Senegalese bird export company and the CITES trade data (Sanz‐Aguilar et al., 2014), this is the general area where these two species have historically been caught for export (and still are). We collected data for the phenotypic characterization of individuals (sex, age, morphometric, and behavioral measures, and a feather to measure corticosterone levels, see below for details). All sampled birds were released in situ (after marking to avoid resampling of the same individual).

We mist netted at two locations where birds were feeding or were moving toward feeding areas. Mist netting involves catching flying birds with nets that are difficult to see when stretched between vertical poles (Figure 1b2), and it is one of the most common methods in the scientific monitoring of passerines (Dunn & Ralph, 2004).

### 2.2 Sampling of individuals entering the bird trade and follow‐up during the first invasion stages

We studied potential selection during three early stages of the original invasion pathway via the international trade of exotic birds by sampling birds caught by Senegalese trappers, and monitoring their fate until international export, usually 1–3 months later (we did not assist in the capture, transport and keeping of these birds). In stage 1, we accompanied professional local bird trappers working for the Senegalese company that exports *P. melanocephalus* and *E. afer* to other countries. Between September 6–13, 2014, they caught individuals using a traditional clap net lying on the ground baited with seeds and stuffed decoys to attract birds (Figure 1b3‐b4) in the same general area as described for the reference sample (NATIVE) above. Trapping was done at two different localities than ours, but sufficiently nearby (about 1 and 30 km away from our localities) that genetic differentiation between and within mist‐netted and traditionally caught samples is absent across 10 microsatellite loci (Mueller et al., 2017), as is to be expected in these common, widespread and mobile species.

We characterized the phenotype of all these individuals in the same way as for the native population (see details below) and marked them with uniquely numbered plastic rings, after which they were handed back to the trappers as fast as possible. Birds were processed with protocols that are standard in the field, and the study design was approved by the relevant national ethical committee (bioethical subcommittee of CSIC‐Spain, project CGL‐2012–3523) following European regulations. All captures (by us and by the Senegalese trappers) were done with permits from the relevant Senegalese authorities.

The first invasion filter of selective uptake was assessed by comparing the traditionally caught birds (UPTAKE, N*_P. melanocephalus_* = 448, N*_E. afer_* = 529) with those caught by us using mist nets (“uptake filter” or UPTAKE‐NATIVE comparison). The presence of such an uptake filter is hypothesized to exist because several studies have suggested that trapping birds with bait and decoys can lead to biases in the captured samples regarding several traits (e.g., Borrás & Senar, 1986; DomèNech & Senar, 1997; Weatherhead & Greenwood, 1981). There is an important caveat though: the extent to which this comparison reflects an actual uptake filter depends on the representativeness of our mist‐netted sample. We take up this issue in the Discussion, but for now, we assume it is sufficiently representative. We then monitored the early survival of the individuals captured by local trappers. All individuals were kept at high densities for about one week in traditional storage cages (Figure 1b6,7) close to the trapping sites, and were then transported 350 km in the same cages (Figure 1b7) to the facilities of the bird‐trading company in Dakar (about seven hours driving on the roof of a bus). Survival was monitored until about one week after arrival in Dakar. Therefore, the second invasion filter where selection could take place was during these 14‐to‐18 days, when individuals either acclimated successfully to early captivity and transport (ACCLyes, N*_P. melanocephalus_* = 235, N*_E. afer_* = 313) or were unable to do so (ACCLno, N*_P. melanocephalus_ = *80, N*_E. afer_* = 133). We thus compared surviving and nonsurviving birds (“acclimation filter” or ACCLyes–ACCLno comparison).

In the last stage we investigated, the remaining birds were communally kept in storage cages (Figure 1b6). According to the Senegalese bird export company, birds are typically stored in these conditions from one to three months before export. Thus, a third invasion filter during which selection was evaluated was this longer‐term survival in captivity. We assessed selection by comparing individuals that survived the first 30 days with those that did not survive (“captivity filter” SURVyes, N*_P. melanocephalus_* = 143, N*_E.afer_* = 175 versus SURVno, N*_P. melanocephalus_* = 92, N*_E.afer_* = 138).

Finally, since selection may act in different directions across sequential filters, we aimed at testing cumulative selection throughout pre‐introduction stages. Cumulative selection was tested by comparing the native population (birds sampled with mist nets) with the population of surviving individuals at the end of the captivity period (SURVyes‐NATIVE comparison).

### 2.3 Characterization of individual phenotypic variation

Just after capture, we took the following morphological measurements from all individuals of the two species: wing length, body mass, external skull dimensions (width, height, and total length including the beak), and beak dimensions (width, height, and length). We used wing length as a proxy of body size, and body mass as a proxy of condition (by statistically controlling for wing length in our models, see below). Since we could not measure brain size directly in live individuals, we used head dimensions as a proxy. Head volume (cm^3^) was calculated as the product of head length (minus beak length), head width and head height. This head volume showed a positive correlation with actual braincase volume of cleaned skulls in our two study species (own data, see Supplementary Information Appendix S1) as well as in another passerine species (Møller, 2010), and we will therefore infer that individuals with larger head volumes have larger brain sizes. We used the beak measurements (width, height, and length) to obtain proxies for beak size and beak shape. For this, we performed a principal component analysis. For both species, the first axis (with roughly equal loadings and equals signs: PC1 *_P. melanocephalus_* = length: 0.77, width: 0.42, height: 0.49, 70% in variance explained and PC1 *_E. afer_* = length: 0.58, width: 0.56, height: 0.59, 64% in variance explained) is interpreted as beak size (higher PC scores indicate larger beaks) and the second axis (with opposite signs: PC2 *_P. melanocephalus_* = length: 0.63, width: −0.66, height: −0.42, 25% in variance explained and PC2 *_E. afer_* = length: 0.81, width: −0.47, height: −0.35, 27% in variance explained) as beak shape (higher PC scores indicate more pointed beaks).

For behavioral characterization, we recorded if individuals pecked at a finger (presented in front of the bird), and if they tried to escape while taking the measurements (two binary scores). This was scored by the same researchers who handled similar sets of birds, so any variation between observers would only add noise to the data, not introduce bias (this is also true for other measurements).

In addition, we collected the two outermost tail feathers and used one of them to quantify feather corticosterone (CORTf) levels. Since circulating corticosterone is deposited in the feather structure as it grows (Bortolotti et al., 2008; Jenni‐Eiermann et al., 2015), quantification of corticosterone levels in feathers (CORTf) has been used as a long‐term biomarker of the energetic expenditure and allostatic load experienced by the individuals during molt (Johns et al., 2018; Will et al., 2014). We used radioimmunoassay (RIA) following Bortolotti et al. (2008) in a stratified random subset (randomly selecting the same number—where possible—of individuals for each combination of sex, age and filter stage) of 283 individuals (*E. afer* *n* = 142, *P. melanocephalus* *n* = 141). Antiserum (C8784; lot 090M4752) and purified corticosterone (C2505, Lot 22K1439) for standards were purchased from Sigma‐Aldrich Chemicals (Saint Louis, Missouri, USA). Feathers (previously kept dry and at room temperature in the dark) were extracted in two extraction batches, and the recovery efficiency (estimated by including feather samples spiked with 5,000 CPM of 3H‐corticosterone) was greater than 94%. Serial dilutions of feather extracts from both species produced displacement curves parallel to the standard curve. Samples were randomly measured in duplicate in five different RIAs. Assay variation was calculated as the % coefficient of variation (CV) resulting from repeated measurement of 6 aliquots of the same standard corticosterone solution in each assay. The coefficient of variation within assay was 5.17% and between assays was 10.38%. The average detection limit (ED 80) was 22.39 (±2.03, *SD*) pg per assay sample, and all the values were above the detection limit. CORTf values are expressed as a function of feather length (pg/mm) following Bortolotti et al. (2008, 2009).

The sex of individuals was determined based on plumage and size characteristics (adults are sexually dimorphic in breeding plumage and, especially *Ploceus*, in size). For uncertain individuals and all individuals in nonbreeding plumage, sex was established by a PCR‐based molecular method following Griffiths et al. (1998), using a small drop of blood taken from all individuals from the wing vein. The age and reproductive stage of individuals were assessed through visual inspection of plumage characteristics (males) and brood patch (females). These species show deferred plumage and sexual maturity, and thus, most of the nonbreeding adults would be one‐year‐old birds, so we divided age into three categories: juveniles, one‐year‐old (adult nonbreeders), and older (breeding) birds.

### 2.4 Data analysis

We used generalized linear models (binomial error distribution; logit link function) and R software (R Core Team, 2017) to test for selection during each studied step of the invasion pathway (“filter”). Models were fitted for each species and each potentially selective filter separately. For the first “uptake filter,” we modeled as response variable the origin of the individuals (uptake/native) and for the next two filters (i.e., “acclimation filter” and “captivity filter”), the response variable was the probability of passing the filter (i.e., individual survival during the filter). In addition, to test for the cumulative selection throughout filters, we used the origin of individuals (native/survivor) as dependent variable for the survivors‐native comparison. The tested effects were sex (male/female), age (reproductive adult, nonreproductive adult or juvenile), wing length, body mass, head volume, CORTf levels (pg/mm), pecking behavior (yes/no), escape behavior (yes/no), beak size (PC1 scores), and beak shape (PC2 scores). All the continuous variables used in the models were standardized (z‐scores, for each species and comparison separately) to allow for a direct comparison of effect estimates. In comparisons where survival is modeled, these estimates equal phenotypic selection gradients (Janzen & Stern, 1998; Lande & Arnold, 1983).

Since models including all variables did not converge, we first fitted a basic model including only sex, age, wing length, and body mass as explanatory variables. To test the other variables, we added these to this basic model (see Table 1). In this way, we fitted separate models for CORTf, behavior (including pecks and escapes in the same model) and head morphology (including head volume, beak size, and beak shape in the same model) (Table 1). No strong correlations were found between the variables tested when controlling for sex and age (all *r* < .34; see Table S1 and S2), justifying such separate models. To test for nonlinear selection, we also fitted models that included the quadratic form of all continuous variables. These multiple regression models provide the relative effects of the variables tested, independent of sex or age (and the other included covariates). However, because the sex or age ratio by themselves may be important for the success of an invasive population, we also fitted simple models with only one unique response variable (sex or age). In all cases, the distributions of residuals were visually assessed to check conforming to statistical model assumptions.

**Table 1:** Overview of the statistical effects of several phenotypic traits on the probability of passing a specific selective filter (uptake, initial acclimation and captivity), as well as all the three filters together (cumulative selection), in two invasive bird species (*Ploceus melanocephalus* and *Euplectes afer*)

Tested variable	Model	*Ploceus melanocephalus*				*Euplectes afer*			
		Uptake	Acclimation	Captivity	Cumulative selection	Uptake	Acclimation	Captivity	Cumulative selection
Sex (male)	Pass ~ basic model	−3.33*	−0.53	0.75	−3.34*	0.83*	−0.47	0.27	0.84*
Age (older birds)	Pass ~ basic model	−0.87*	0.15	−0.71	−0.89*	−0.14	0.08	−0.45	−0.29
Age (juveniles)		−0.72*	1.30*	−0.21	−0.34	−0.69*	0.55	−0.05	−0.39
Wing length	Pass ~ basic model	1.39*	−0.25	−0.36	1.07*	0.15	−0.10	−0.08	0.12
Body mass	Pass ~ basic model	1.08*	0.44	0.45	1.42*	−0.36*	1.04*	0.27	0.05
Head volume	Pass ~ basic model + **head volume** + **beak size** + **beak shape**	−0.60*	**0.47**	0.22	−0.55*	−0.03	**0.31**	−0.08	0.02
Beak size (PC1)		0.48*	−0.02	0.20	0.49*	0.23*	−0.12	−0.04	0.18
Beak shape (PC2)		−0.47*	−0.15	0.08	−0.48*	−0.06	0.07	−0.05	−0.01
Feather Corticosterone	Pass ~ basic model + **corticosterone**	1.24*	−0.48	−0.03	1.06*	1.09*	−0.25	−0.48	**0.68**
Pecks (yes)	Pass ~ basic model + **pecks** + **escapes**	−1.55	−0.08	1.19	−0.53	−0.06	−1.37	**−1.35**	−0.72
Escapes (yes)		−0.49	0.94	0.19	−0.26	−0.58*	1.30*	0.29	−0.27

Note

The model used and the estimated coefficients are provided (basic model: sex + age class + wing length + body mass); in bold when significant (*p* < .05) and underlined when a trend was observed (0.05 < *p* < .10).

Asterisks indicate a significant effect after adjusting for a false discovery rate (q‐value <0.05) (Standard errors are provided in Table S3).

Continuous variables were standardized so reported effect estimates can be directly compared.

Running several models for several species and several filter stages to test what is basically the same hypothesis increases the risk of obtaining false positives due to multiple testing. To test the general hypothesis of whether there is selection in the early stages of biological invasion, we combined p‐values of all the fitted effects by Fisher's method (Rosenthal, 1978), which provides a single p‐value for the overall pattern, independent of the number of tests performed. Additionally, we adjusted the significance of the individual effects (*p*‐values) for false discovery rate (Verhoeven et al., 2005) across the total set of 88 comparisons (most conservative approach), to appropriately reduce type I errors. Both approaches assume that test results are independent, and this is approximately true given that explanatory variables are mostly poorly correlated (see Tables S1 and S2: correlations range from −0.33 to 0.33).

## 3 RESULTS

We first provide general results. For the first filter (uptake), we observed significant effects in almost all (9 out of 11) of the phenotypic comparisons tested in *P. melanocephalus*, and in the majority of comparisons (6 out of 11) in *E. afer* (Table 1). For the second and third filter (acclimation and captivity), progressively fewer effects reached statistical significance, but in total 21 comparisons reached significance and an additional 6 near‐significance (out of 66 comparisons, uncorrected for multiple testing). When combining the p‐values of all the previous comparisons (by Fisher's method), we found that selection was significantly acting during the studied pre‐introduction stages (χ^2^ = 765.6, *p* < .0001). Cumulatively, selective filters changed the mean of the traits in the surviving individuals for 8 comparisons in *P. melanocephalus* and 2 comparisons in *E. afer*. We did not find significant effects of quadratic variables (not shown), so we do not further consider nonlinear (incl. stabilizing and disruptive) selection.

**Figure 2:**
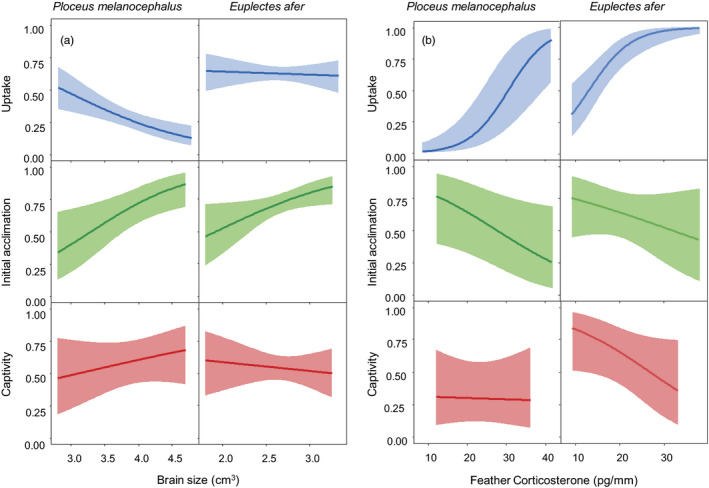
Statistical effects of (a) head volume and (b) feather corticosterone levels on the probability of progressing across the three selective filters studied (uptake, initial acclimation, and captivity) for *Ploceus melanocephalus* and *Euplectes afer*. Lines represent model predictions (corrected for covariates, see Table 1), and shadow areas represent 95% confidence intervals

We now describe the principal patterns observed. For both species, traditionally trapped individuals had smaller relative head volumes than mist‐netted individuals, but individuals with relatively larger head volumes were more likely to survive during the next filter of initial acclimation (Table 1, Figure 2a). A similar reversed pattern was revealed for CORTf levels in both species: traditionally trapped individuals had higher corticosterone levels than mist‐netted individuals, but survival during initial acclimation and captivity was higher for individuals with lower hormone levels (Table 1, Figure 2b). The cumulative effect was that surviving individuals displayed higher levels of CORTf than those captured with mist nets (Table 1). The effect of sex (corrected for other covariates, so “relative sex”; see further below for the potentially more relevant results for uncorrected sex) differed between species: in *P. melanocephalus,* fewer males were trapped with traditional methods compared to the mist‐netted sample, whereas in *E. afer* more males were trapped traditionally. Both effects were transferred to the corresponding surviving populations at the end (Table 1). Age effects were similar in both species: older adults and juveniles showed lower probabilities to be trapped traditionally than one‐year‐old adults, and juveniles were more likely to survive during the initial acclimation (Table 1). The final surviving population of *P. melanocephalus* held a lower proportion of older adults than present in the mist‐netted sample (Table 1). The wing length of the individuals had an effect on uptake, with larger individuals being more likely to be trapped in both species, resulting in larger surviving individuals of *P. melanocephalus* at the end than in the mist‐netted sample (Table 1). Heavier *P. melanocephalus* individuals were more likely to be trapped and this effect remained at the end. However, for *E. afer* this effect was the opposite, and heavier individuals showed higher survival probabilities in the initial acclimation filter (Table 1). In both species, trapped individuals had bigger beaks than mist‐netted samples, and especially in *P. melanocephalus* also a more pointed beak, for which these effects are also present at the end. Behavioral traits were also selected upon during the filters, especially for *E. afer*, where individuals which attempted to escape during manipulation were less likely to be trapped and showed a higher survival probability during initial acclimation, while individuals displaying pecking behavior showed a lower survival probability during the captivity filter.

Models fitted to test the overall effect of sex or age (with no other controlling variables) showed that more males were trapped for both species, and that *P. melanocephalus* males had a higher survival during the captivity filter. The cumulative selection resulted in more males in both species (Table 2). With respect to age, juveniles of both species were less trapped, and for *P. melanocephalus,* juveniles had a higher survival during acclimation (Table 2). Older adult birds had lower survival in captivity in *P. melanocephalus,* and they had higher survival during initial acclimation in *E. afer*. The cumulative selection resulted in fewer juveniles in *P. melanocephalus* (Table 2).

**Table 2:** Overview of the statistical effects of sex and age (without controlling for other variables) on the probability of successfully passing a specific selective filter (uptake, initial acclimation and captivity), and all the three filters together (cumulative selection), in two invasive bird species (*Ploceus melanocephalus* and *Euplectes afer*)

Tested variable	Model	*Ploceus melanocephalus*				*Euplectes afer*			
		Uptake	Acclimation	Captivity	Cumulative selection	Uptake	Acclimation	Captivity	Cumulative selection
Sex (male)	Pass ~ sex	**0.92**	−0.44	**0.80**	**0.97**	**0.72**	0.44	0.32	**0.98**
Age (older birds)	Pass ~ age	−0.27	0.22	**−0.80**	−0.39	−0.06	**0.45**	−0.19	−0.03
Age (juveniles)		**−1.11**	**1.40**	−0.44	**−0.80**	**−0.67**	0.57	−0.01	−0.46

## 4 DISCUSSION

Our results clearly demonstrate the existence of selective filters acting before the actual introduction of invasive species, and affecting a wide range of phenotypic traits. Depending on the trait, selection may act similarly or differently among filters and species (Table 1). This demonstrates that selective filters could have independent effects, depending on the invasion stage (and its conditions) and the species on which they act. Therefore, punctual and cumulative (net) selection on the individual characters of potential invaders can no longer be assumed to be absent during the early stages of a biological invasion (Briski et al., 2018; Carrete et al., 2012; Chapple et al., 2012).

### 4.1 Potential causes and consequences of pre‐introduction selection on different traits

In general, we do not know in detail why selection acted the way it did, if and how our tested variables are causally involved, and what longer‐term consequences this selection might have, since our study was not designed to answer these further questions. However, we provide some discussion and suggestions for future tests and encourage subsequent research on this.

### 4.2 Brain size and corticosterone

In the uptake filter, we observed that individuals with smaller head volumes and presumably smaller brains were more likely to be caught, potentially reflecting variation in cognitive abilities and escape strategies (Samia et al., 2015). However, in the following stages selection favored larger brains, which may provide a better coping ability with novel situations (Sol et al., 2005) that ultimately increases the probability of survival. This might have an impact on the population invasive potential, since bigger brains facilitate the colonization of variable habitats (Fristoe et al., 2017). Selection acted similarly regarding corticosterone levels: the phenotypes showing higher CORTf titers were more likely to be captured from the wild, but they subsequently experienced lower survival in captivity. Elevated CORTf levels have been used as a biomarker of burden, indicating higher allostatic loads (Johns et al., 2018; Will et al., 2014). The traditional capture methods used by Senegalese trappers likely attracted individuals with higher energy demands, more ready to face risks to obtain food from the baited traps (relative to mist‐netted individuals and probably the general population). Once these birds entered, the captive environment food was provided ad libitum but the competition and levels of exposure to novel and challenging stimuli increased, reducing the survival of the phenotypes showing elevated CORTf levels and suggesting a competitive disadvantage of individuals with higher allostatic loads (see, e.g., Harms et al., 2014; Koren et al., 2012).

### 4.3 Behavior, bill size and shape, body size, and body mass

We also found selection on behavioral traits, suggesting that behavioral (incl. personality) variation may affect coping with unnatural novel situations during the studied invasion stages. This effect may carry over to the coping with future novel situations in the new area of introduction. One of these new conditions is the food source, and in addition to behavior (Sol et al., 2011), beak morphometry can play a role in how individuals deal with a new type of food. In this context, we found that beak morphometry (both size and shape) was selected upon in the capture filter. Moreover, we found that individuals were selected by wing length in the capture filter, with larger individuals being more likely to be trapped in one species. Organismal size is one of the most important determinants of ecology (Begon et al., 2009), so the fact that there was cumulative selection on size should be suspected to have repercussions further along the invasion pathway, both for invasion success and for invasion impact. A higher individual condition (relative body mass) was generally favored while the birds were in captivity, which seems logical when individuals need to cope with new environments and new types of food, and might also have effects further along the invasion.

### 4.4 Sex and age

Finally, sex and age biases can have a great effect on the reproduction rate and thereby on the survival and the expansion of the population that is introduced. Sex showed opposed effects in the composite models depending on the species considered (Table 1), but this is the effect of sex when adjusted for other variables, and rather hard to interpret. When we just observe the total sex ratio (without controlling for other variables like size, which differs between the sexes), more males were trapped for both species (Table 2). Likewise, fewer juveniles were trapped. Generally, these effects were maintained until the end of our study. Since both species are polygynous and have a delayed age of first breeding (Craig, 1980; Habig et al., 2017), the population reproductive rate is expected to be mostly determined by the number of reproductive adult females. In this case, the observed pre‐introduction selection against females could reduce establishment success and the expansion rate of the introduced population. On the other hand, the observed pre‐introduction selection against juveniles might increase invasion potential (more adult breeders).

### 4.5 What does the uptake filter reflect?

We have so far assumed that the sample we caught with mist nets reflected the composition of the native population, and have interpreted that the difference between this sample and that caught by the trappers reflects an uptake filter. Individuals caught with traditional clap nets are actively attracted to the catching site with food and decoys, and individuals need to decide whether or not to come down to the ground to feed in order to be caught. This chain of events opens up the possibility that the captured sample is a biased subset of the total population, for example involving individuals with a greater need for food or that are less shy. This has indeed been found in several studies (e.g., Biro & Dingemanse, 2009; Borrás & Senar, 1986; DomèNech & Senar, 1997; Dufour & Weatherhead, 1991; Weatherhead & Greenwood, 1981). In contrast, mist netting—when used in the absence of attractants like food or sound (our case) and in unpredictable sites—can be seen as the passive capture of birds as they move through the environment, so the potential for catching a biased subset of the population appears to be much smaller. However, biases may still enter mist‐netted samples, mostly because of when and where the nets are placed (reviewed in Ralph, Dunn, Peach & Handel, 2004). Locations for trapping with mist nets tend to be selected by researchers for their favorable local conditions, for example, the presence of a dark background to reduce the visibility of the nets, high densities of birds, or simply good access in difficult terrain. Nets may also be removed during times with little bird activity. These considerations also played a role in this study: we selected sites and times of the day with high numbers of active birds and good opportunities to catch. This could result in biases, if the traits we measured covary with how active the individuals are at the times and places where we were catching. This is difficult to fully exclude. Nonetheless, some studies that have compared biases among capture techniques have argued that mist netting is the less biased technique (Borrás & Senar, 1986; DomèNech & Senar, 1997; Weatherhead & Greenwood, 1981), and Simons et al. (2015) reported that there was no evidence that a fraction of their population of House sparrows remained uncaught when using mist nets. Based on these considerations and field experience, mist netting is often the method of choice for the scientific monitoring of passerines (Dunn & Ralph, 2004). One bias that Simons et al. (2015) and others (e.g., DomèNech & Senar, 1997) do report is that some individuals may learn to avoid (the locations with) mist nets after their first capture. However, this does not affect our study since we only use individuals captured for the first time. Taking all of this into account, and as we cannot be completely sure about the extent to which our mist‐netted sample is actually representative of the native population, the interpretation of the uptake filter and cumulative selection is also less than perfect. Future studies on uptake filters in other study systems might be able to characterize the native population with a greater (perfect) degree of certainty. Independent of this weakness in our study, we also found evidence for phenotypic selection during the acclimation and activity filters following uptake, and these results are not affected by the selectivity (if any) of mist nets, so our general conclusion that pre‐introduction selection occurs remains unaffected.

### 4.6 Pre‐introduction selection is expected to be general with respect to species, traits, and type of invasion

As described above, it is likely that the phenotypic traits on which pre‐introduction selection acts have relevance for how individuals cope further down the invasion pathway and in newly colonized areas. Therefore, this selection likely shapes the future establishment success, invasiveness, and impact of the introduced population, and there is no reason to think why this should not be generally applicable to any species (Briski et al., 2018; Colautti & Lau, 2015; Zeng et al., 2015). The effects of such early selection on population composition can be varied: it can change the mean phenotypic values of a population (as in this study), but it could also change its variability due to directional or nonlinear selection. The effect of variability on invasiveness (even if the mean is little altered) should not be neglected, since variability itself is an important component of colonization success (Cote et al., 2010; Forsman et al., 2012). In contrast, loss of variation due to one filter decreases the probability that a subset of individuals is pre‐adapted to the next filter (to the extent that this filter is dissimilar to the previous one), and ultimately to the new environment of introduction. These arguments are valid and relevant when this variation is only phenotypic, but additional longer‐term (evolutionary) effects are expected when phenotypic variation has a heritable basis (as is the case for most variable traits; see Mueller et al., 2017 and Mueller et al., 2014 for pre‐introduction selection on genetic variation associated with behavior in *Euplectes afer*).

Our study was framed within the context of the international pet trade, which is acknowledged as an increasingly important source of biological invasions (Abellán et al., 2016). However, early selection should also be expected in other types of invasions, for example, unintentional ones, where nonrandom uptake and survival during transport (e.g., in ships, containers) can be easily imagined (Blackburn et al., 2011; Chapple et al., 2012). Until now, studies assessing how populations undergo micro‐evolutionary changes during biological invasions have exclusively focused on the later stages of invasion (e.g., Blackburn et al., 2009), which have been shown to be very important for the potential impacts (Faillace and Morin, 2016). However, as our results suggest, introduced populations may have already undergone micro‐evolutionary changes (assuming traits are heritable) through selective filters (even when these are artificial and human‐induced) before reaching the establishment stage, and this likely affects all subsequent changes involved in the adaptation to a new non‐native area in the subsequent stages. Similarly, some reintroduced populations from conservation programs have been exposed to (human‐induced) selective conditions in captivity, causing shifts in phenotypic traits that may be critical for reintroduction success (Grueber et al., 2017; McDougall et al., 2006). The selective effects of captivity on behavior (Carrete & Tella, 2015; McDougall et al., 2006), stress physiology (Cabezas et al., 2013), or morphology are well known, causing differences with respect to wild populations (Courtney Jones et al., 2017). Such undesigned selection is also occurring during domestication, causing changes in morphology, behavior, and physiology in many species (Campbell et al., 2015; He et al., 2014). In yet another analogue, hunting may change the structure of populations through a selection of individuals with certain phenotypic characteristics (Berger et al., 2001; Festa‐Bianchet, 2003; Madden & Whiteside, 2014). During the first stages of a biological invasion, human‐induced selection acting during uptake and continuation along the invasion pathway was therefore predicted to affect a range of traits (Briski et al., 2018; Carrete et al., 2012; Chapple et al., 2012) and finally confirmed in this comprehensive study. Further investigation of selection during these virtually ignored early stages of invasion is urgently needed, and should include how early selection influences the invasion success and the impacts of introduced populations, in order to better understand and hopefully effectively manage biological invasions.

## CONFLICT OF INTERESTS

None declared.

## AUTHOR CONTRIBUTIONS

MC, JLT, JB, JP, MSD, and PE designed the study; ABV, MC, JB, JP, CC, MSD, and PE collected data and samples; TM and SC analyzed samples; and ABV and PE analyzed the data and wrote the paper, with input from all other authors.

## Supporting information

Appendix S1

## Data Availability

Data for this study are available at, Selection on individuals of introduced species starts before introduction, Dryad, Dataset, https://doi.org/10.5061/dryad.msbcc2fww (Baños‐Villalba et al., 2020).
