# Supplementary material for: Selection on individuals of introduced species starts before the actual introduction
Source: Evol Appl. 2020 Dec 15;14(3):781–93. doi: 10.1111/eva.13159 (PMC7980263; doi:10.1111/eva.13159)
Supplement: Supplementary file 1 — Appendix S1 [file EVA-14-781-s001.doc]

*Supporting Information for:*

Selection on individuals of introduced species

starts before introduction

**This file includes:**

- Fig. S1
- Tables S1 - S3

Fig. S1. Relationship between head volume estimated in vivo (head length * head width * head height) and brain case volume estimated post-mortem in cleaned skulls (mean of two measures of the mass of sand poured into the brain case). Red dots depict *Euplectes afer* (R= 0.201) and blue dots *Ploceus melanocephalus* (R=0.844).

**Table S1. Below diagonal: average correlation coefficient among the measured phenotypic traits (raw data) for the 6 different groups (all possible combinations of sex (male/female) and age (juveniles, one year old, older birds)) in *Ploceus melanocephalus.* Above diagonal: avarage p-value of these correlations.**

|  | Body mass | Wing length | Head volume | Feather  corticosterone | Pecks | Escapes | Beak size | Beak shape |
| --- | --- | --- | --- | --- | --- | --- | --- | --- |
| Body mass |  | 0.000 | 0.293 | 0.604 | 0.196 | 0.470 | 0.019 | 0.417 |
| Wing length | **0.33** |  | 0.261 | 0.420 | 0.052 | 0.557 | 0.084 | 0.570 |
| Head volume | **0.12** | **0.12** |  | 0.566 | 0.559 | 0.451 | 0.305 | 0.001 |
| Feather corticosterone | **-0.02** | **-0.06** | **-0.04** |  | 0.503 | 0.579 | 0.470 | 0.661 |
| Pecks | **0.04** | **0.01** | **0.05** | **-0.11** |  | 0.004 | 0.270 | 0.555 |
| Escapes | **0.01** | **-0.04** | **0.03** | **-0.03** | **0.27** |  | 0.578 | 0.230 |
| Beak size | **0.26** | **0.20** | **-0.08** | **0.03** | **-0.10** | **0.06** |  | 0.430 |
| Beak shape | **-0.02** | **-0.01** | **-0.33** | **0.01** | **0.02** | **-0.13** | **-0.02** |  |

**Table S2. Below diagonal: average correlation coefficient among the measured phenotypic traits (raw data) for the 6 different groups (all combinations of sex (male/female) and age (juveniles, one year old, older birds)) in *Euplectes afer.* Above diagonal: avarage p-value of these correlations.**

|  | Body mass | Wing length | Head volume | Feather  corticosterone | Pecks | Escapes | Beak size | Beak shape |
| --- | --- | --- | --- | --- | --- | --- | --- | --- |
| Body mass |  | 0.026 | 0.006 | 0.707 | 0.573 | 0.409 | 0.059 | 0.554 |
| Wing length | **0.25** |  | 0.391 | 0.481 | 0.411 | 0.459 | 0.020 | 0.644 |
| Head volume | **0.29** | **0.11** |  | 0.558 | 0.724 | 0.329 | 0.382 | 0.059 |
| Feather corticosterone | **-0.10** | **-0.02** | **-0.12** |  | 0.164 | 0.456 | 0.507 | 0.392 |
| Pecks | **0.004** | **0.01** | **0.04** | **0.08** |  | 0.058 | 0.376 | 0.508 |
| Escapes | **0.07** | **0.09** | **-0.05** | **-0.02** | **0.24** |  | 0.343 | 0.390 |
| Beak size | **0.22** | **0.28** | **-0.02** | **0.05** | **-0.07** | **0.13** |  | 0.332 |
| Beak shape | **0.05** | **0.02** | **-0.26** | **-0.22** | **0.04** | **0.01** | **0.005** |  |

**Table S3. Overview of the standard errors of the effects (shown in table 1) of several phenotypic traits on the probability of passing a specific selective filter (uptake, initial acclimation and captivity), as well as all the three filters together (cumulative selection), in two invasive bird species (*Ploceus melanocephalus* and *Euplectes afer*).**

|  |  | | |  | | | | | ***Ploceus melanocephalus*** | | | | | | | | ***Euplectes afer*** | | | | | | |
| --- | --- | --- | --- | --- | --- | --- | --- | --- | --- | --- | --- | --- | --- | --- | --- | --- | --- | --- | --- | --- | --- | --- | --- |
| **Tested effect** | | **Model** | | | **Uptake** | | **Acclimation** | | | **Captivity** | | **Cumulative selection** | | **Uptake** | | **Acclimation** | | | **Captivity** | | **Cumulative**  **selection** |  | |
| **Sex** (male) | | | Pass ~basic model | | | 0.446 | | 0.775 | | | 0.772 | | 0.637 | | 0.181 | | | 0.329 | | 0.344 | 0.244 | |  |
| **Age** (older birds) | | | Pass~basic model | | | 0.216 | | 0.339 | | | 0.385 | | 0.305 | | 0.165 | | | 0.276 | | 0.288 | 0.220 | |  |
| **Age** (juveniles) | | |  |  |  | 0.193 | | 0.405 | | | 0.370 | | 0.264 | | 0.208 | | | 0.398 | | 0.383 | 0.281 | |  |
| **Wing length** | | | Pass~basic model | | | 0.217 | | 0.354 | | | 0.384 | | 0.291 | | 0.087 | | | 0.157 | | 0.160 | 0.115 | |  |
| **Body mass** | | | Pass~basic model | | | 0.186 | | 0.336 | | | 0.341 | | 0.255 | | 0.082 | | | 0.164 | | 0.156 | 0.111 | |  |
| **Head volume** | | | Pass~ basic model + **head volume** +  **beak size** + **beak shape** | | | 0.132 | | 0.209 | | | 0.216 | | 0.182 | | 0.088 | | | 0.145 | | 0.147 | 0.114 | |  |
| **Beak size (PC1)** | | |  |  |  | 0.131 | | 0.253 | | | 0.251 | | 0.175 | | 0.078 | | | 0.147 | | 0.145 | 0.105 | |  |
| **Beak shape (PC2)** | | |  |  |  | 0.091 | | 0.147 | | | 0.147 | | 0.130 | | 0.075 | | | 0.130 | | 0.131 | 0.101 | |  |
| **Feather Corticosterone** | | | Pass~ basic model + **corticosterone** | | | 0.276 | | 0.252 | | | 0.296 | | 0.324 | | 0.279 | | | 0.251 | | 0.282 | 0.296 | |  |
| **Pecks** (yes) | | | Pass~ basic model + **pecks**  + **escapes** | | | 0.808 | | 1.185 | | | 1.246 | | 1.355 | | 0.330 | | | 1.077 | | 0.600 | 0.370 | |  |
| **Escapes** (yes) | | |  |  |  | 0.360 | | 0.552 | | | 0.628 | | 0.544 | | 0.196 | | | 0.421 | | 0.322 | 0.260 | |  |
